# Circular RNA expression alteration and bioinformatics analysis in patients with acute cerebral infarction injury

**DOI:** 10.1080/21655979.2021.2009960

**Published:** 2021-12-07

**Authors:** Xiao-Lei Hu, Qian Su, de-Long Meng, Yuan-Shui Ren, Zhi-Qiang Su

**Affiliations:** a Department of Neurology, The First Affiliated Hospital of Harbin Medical University, Harbin, Heilongjiang China; b ICU, The First Affiliated Hospital of Harbin Medical University, Harbin, Heilongjiang China; c Department of Neurology, Jiaozuo Second People’s Hospital, Jiaozuo, Henan China

**Keywords:** Acute cerebral infarction, circRNAs, bioinformatics analysis, ischemic stroke

## Abstract

In recent years, a steady increase has been detected in the incidence of acute cerebral infarction (ACI). ACI is caused by blood flow disruption, leading to high disability and mortality rates. Understanding the underlying molecular mechanisms is critical toward developing effective therapeutic approaches. Circular RNAs (circRNAs) are an important class of non-coding RNAs, which have been implicated in several molecular pathways, and their dysregulation has been described in several disease conditions. Here, we set out to explore the possible regulatory role of circRNAs in ischemic stroke and study their molecular function in disease. First, we applied high-throughput sequencing techniques to identify the differential changes of plasma circRNAs expression in patients with acute cerebral infarction. Next, we used GO and KEGG pathway analysis to predict the function of differentially expressed circRNAs. Moreover, we have assessed the possible interaction between the identified differentially expressed circRNAs and miRNAs. Finally, we have selected and validated five downregulated circRNAs by RT-qPCR. Together, the results of this study provide evidence that circRNAs are potential biomarkers for early diagnosis of cerebral infarction and have to be considered as targets for drug treatment.

## Introduction

Acute cerebral infarction (ACI) is one of the most common and high-incidence cerebrovascular diseases, accounting for approximately 70% of all stroke [1]. ACI is most frequently caused by hypoxia and ischemia of brain tissue, leading to a nerve function damage [2]. Currently, our understanding is rather limited regarding the molecular mechanisms leading to the development of ACI, and we mainly rely on imaging and hemodynamics to evaluate the condition and prognosis [3–5]. Therefore, better understanding of disease-associated changes at a molecular level is necessary in order to find potential intervention or treatment targets.

Circular RNAs (circRNAs) are a highly conserved and stable class of non-coding RNAs, which are found in plant as well as animal cells [6]. CircRNAs have tissue specificity, and they have been described in the regulation of gene expression. Moreover, multiple studies have shown that circRNAs can play a specific role in disease development and progression [7,8]. Mechanistically, circRNAs can enter the blood with exosomes as carriers and stably exist in the peripheral blood. Because it can stably exist in the collected blood samples, the information of tissues and cells can be obtained by detecting the blood samples [9]. In addition, exosomes can cross the blood-brain barrier. Since circRNAs are released from the brain and their presence can be detected in peripheral blood, circRNAs are expected to be suitable biomarkers for auxiliary diagnosis of diseases. In fact, detection of circRNAs would provide an opportunity for a noninvasive examination and early diagnosis of ACI from easily accessible peripheral blood samples.

Here, we hypothesize that some circRNAs are differentially expressed in the body after ACI, and these circRNAs may play an important role in regulating gene expression and influence the disease progression by influencing some specific signal pathways. In order to explore the relationship between circRNAs and ACI, we first quantified the differential changes of plasma circRNA expression in patients with ACI by a high-throughput sequencing technique. The function of differentially expressed circRNAs was predicted by bioinformatics analysis, including GO and KEGG pathway analysis. The possible interaction between circRNAs and miRNAs was explored by predicting their binding. In addition, five downregulated circRNAs were selected for RT-qPCR verification. The purpose of this study is to reveal the regulatory role of circRNAs during ischemic stroke and their interaction with miRNAs by analyzing the expression profile of circRNAs after ischemic stroke. Overall, the results of this study reveal that developing circRNAs are potential novel markers for early diagnosis of cerebral infarction and they should be considered as drug treatment targets.

## Materials and methods

### Patients

We have collected samples from patients diagnosed with ACI after their admission to the First Affiliated Hospital of Harbin Medical University. Sample collection took place between July 2020 and September 2020. Patients admitted to the hospital due to weakness of one limb were considered for this study. In total, after patients met with all of our inclusion/exclusion criteria (see below), we have been able to collect samples from a total of 3 patients, including two males and one female, with an average age of 61.3 years. Patients had no obvious systemic diseases, except hypertension and diabetes. All patients had clear symptoms of neurological deficits. MRI or CT confirmed the diagnosis of ischemic stroke [10]. In addition, three patients with normal physical examination at the health management center were selected as the normal control group, including one male and two females, with an average age of 61 years.

## Inclusion criteria

[1] Meet the general diagnostic criteria of ACI and have exact symptoms of focal neurological deficit, and the diagnosis of ischemic stroke is confirmed by brain MRI (Supplementary Figures 1–3) or CT.

[2] The age is between 55 and 70 years old, and it may be complicated with hypertension and/or diabetes.

[3] The onset time is less than 7 days

## Exclusion criteria

1. Age ≤55 years old or ≥70 years old;
2. The onset time is more than 7 days; Have a history of other nervous system-related diseases except hypertension and diabetes.

[2] Imaging suggests other intracranial lesions, such as hemorrhage and space occupation.

[3] It is complicated with respiratory, circulatory, blood, and other system diseases or has a history of corresponding diseases in the past.

## Sample preparation

After the patient was admitted to hospital, the fasting venous blood of the patient was collected in the morning. Then, plasma was separated by centrifugation, stored in EP tube, labeled, and stored in an ultra-low temperature refrigerator at −80°C until further downstream processing.

## Preparation, quality control, and sequencing of RNA library

The qualified samples were enrolled into the group, and the total RNA was extracted after DNA and protein removal. RNA concentrations extracted from samples were measured using a Nano Drop 1000ND analyzer (Thermofisher Scientific, Waltham, MA, USA). The ratio of OD260/OD280 was used as the index of RNA purity. Only samples with OD260/OD280 ratio between 1.8 and 2.1 were considered for downstream processing. A circRNA sequencing library was prepared according to the following steps..

1. RNA pretreatment. 5 μg of total RNA was used as starting material, and the circRNA enrichment kit (Cloud-SEQ Inc, Shanghai, China) was used for the enrichment of circRNA to obtain the expression profile of circRNAs.
2. RNA library construction. RNA was pretreated by TruSeq stranded total RNA library prep kit (Illumina, USA). RNA sequencing library was prepared according to the manufacturer's recommendations.
3. RNA integrity quality control: We have assessed RNA and library quality and quantification using a BioAnalyzer 2100 instrument (Agilent Technologies, USA). In brief, 10 pM library was transformed into single-stranded DNA molecules, which were sequenced using an Illumina 4000 HiSeq sequencer and paired end mode.

## Functional go analysis of differential circRNAs

At the time of this study, no special annotation information was available for circRNAs, so we utilized Yunxu organisms to carry out GO functional analysis on the host genes that were found to be linked to differentially expressed circRNAs. GO hits with a p-value <0.05 were considered as statistically significant.

## Analysis of KEGG pathway of genes derived from different circRNAs

According to the detected difference in the expression of circRNAs, we carried out a pathway analysis. P-values for each path with different genes (circRNA-derived genes) are indicated. By analyzing the pathways of differentially expressing circRNA-derived genes, we can infer their involved pathways and their biological functions. A P value <0.05 is used as the threshold of significant enrichment.

## Prediction of interaction between CircRNAs and microRNA

Previous studies indicated specific interactions between differentially expressed circRNAs and miRNAs, suggesting that circRNAs may function as miRNA sponges. We utilized miRNA target gene prediction software TargetScan (version V7.0) to explore the function of circRNAs. Moreover, miRNA prediction software independently developed by Yunxu Biotechnology Co., Ltd. based on miRanda and TargetScan was also used to predict the binding sites of miRNAs target genes.

## Extraction of total RNA from samples

First, whole blood was homogenized after thawing. In brief, 0.2 ml of chloroform (Shanghai Shenggong) was added to every sample homogenized in 750 μl of TRIzol LS Reagent. After shaking the tube vigorously by hand for 15 seconds, samples were incubated at room temperature for 2 to 3 minutes. Next, we centrifuged the samples at 12,000 × g for 15 minutes at 4°C. After centrifugation, the mixed liquid was divided into the lower red phenol-chloroform phase, the middle layer and the upper colorless water phase. The waterish phase, consisting the RNA was transferred to a new centrifuge tube, and 500 μl of isopropanol (Shanghai Shenggong) was added to each sample. After mixing well, samples. Were incubated at 15 to 30°C for 10 minutes, then centrifuged at 12,000 × g at 4°C for 10 minutes, and form on the bottom and side walls of the tube Gelatinous precipitation block. RNA pellets were washed with ethanol and centrifuged again at 7,500 × g at 4°C for 5 minutes. After removing the ethanol, samples were dried on air for 5–10 minutes.Finally, RNA was dissolved by adding RNase-free water to dissolve RNA. The obtained RNA solution was stored at −70°C. The corresponding values at 260 nm and 280 nm were detected by Nano Drop ND 2000 spectrophotometer (Thermo Fisher Scientific, Waltham, MA, USA) to calculate the total amount and quality of RNA.

Different specifications of EP tubes, pipettes, and other experimental consumables used in the process of extracting total RNA all use RNase-free disposable consumables (Shanghai Shenggong), and the operation is carried out on a clean bench icebox. Equipment used in the experiment, such as tweezers, was sterilized at high temperature and high pressure for 2 hours.

## Statistical method

Data analysis was carried out using Microsoft Excel (2019). Briefly, the relative expression level of circRNAs was reflected by calculating the relative expression level of target circRNAs in the diseased group compared with the control group. The experimental data were statistically analyzed by SPSS 17.0. Differences with a P < 0.05 was considered statistically significant.

## Results

### Expression pattern of cyclic ribonucleic acid after acute cerebral infarction

In this study, we set out to investigate the role of circRNAs in ACI. In order to find new biomarkers and therapeutic targets for ACI, we studied the differential changes of plasma circRNAs expression in patients with ACI using high-throughput sequencing techniques.

**Figure 1.**
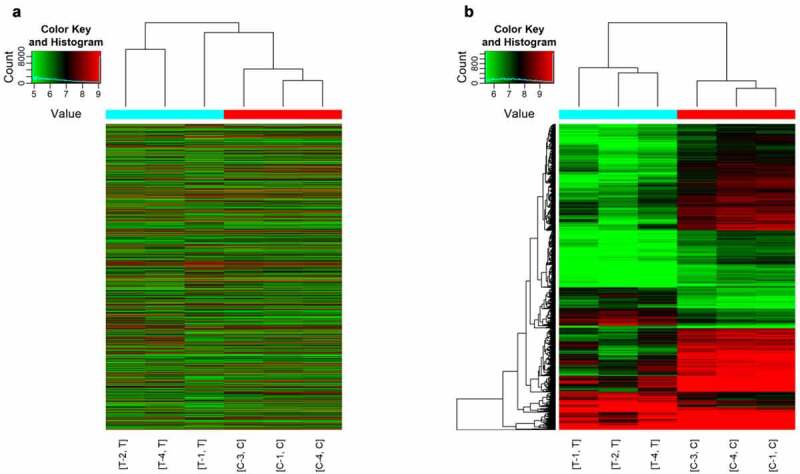
Hierarchical clustering of circRNAs expression. C1/3/4 refers to the peripheral blood samples of sham control group, and T1/2/4 refers to the peripheral blood samples of SCI group. (a) Hierarchical cluster analysis included 13,279 circRNAs in ACI group and sham control group. (b) Hierarchical cluster analysis includes differentially expressed circRNAs (P < 0.05; folding change≥2). There was significant difference between ACI group and sham operation group

**Figure 2.**
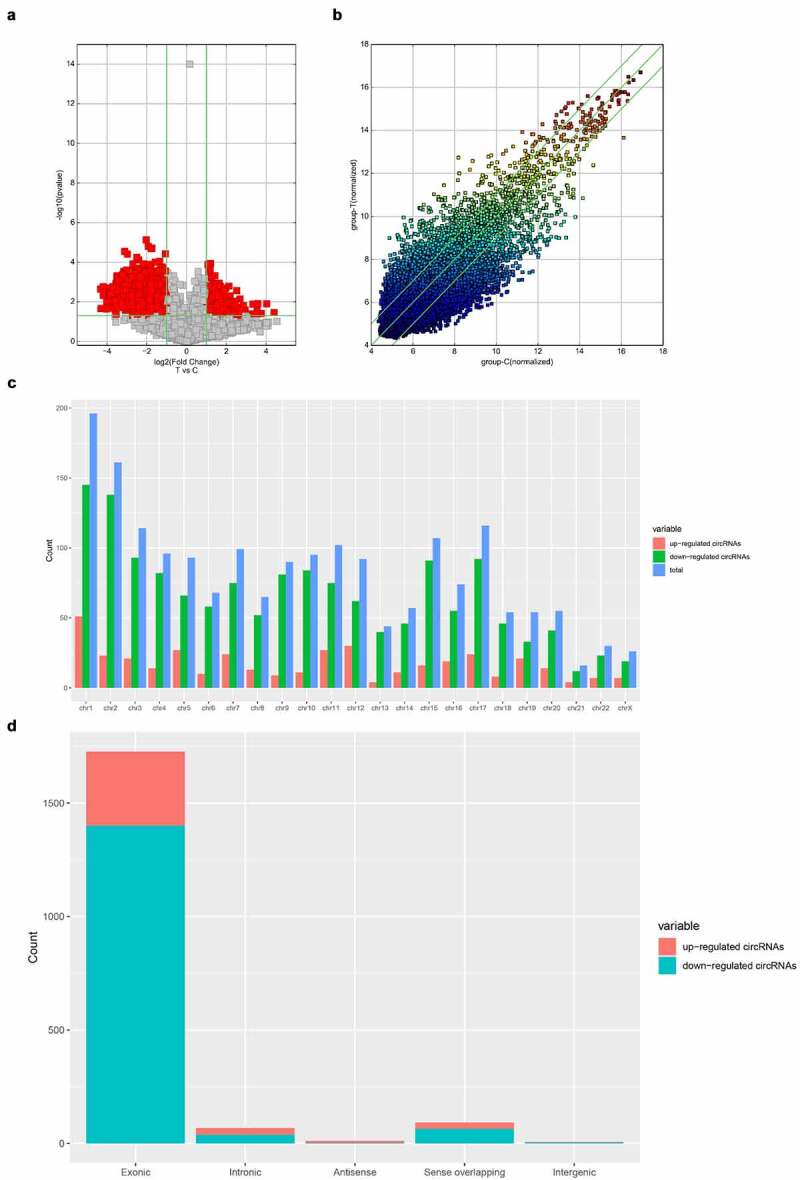
Difference of expression profiles of circRNAs between two groups. (a) Scatter plot show the difference of circRNAs expression between sham operation group and ACI group. The x-axis and y-axis values in the scatter plot are the normalized signal values of samples (log-2 scale) or the average normalized signal values of sample groups (log-2 scale). The green line is the fold change line. The circRNAs above and below the green line represent those circRNAs which show at least 2-fold changes in expression. (b) Volcano map show that cyclic ribonucleic acid was differentially expressed with statistical significance (P < 0.05; folding change ≥ 2). (c) The distribution of circRNAs indicated that the dysregulated circRNAs originated from each chromosome. (d) The classification bar chart of circRNAs based on gene source shows that most cyclic ribonucleic acids changed after ACI are exons

As shown in Figure 1, a total of 1904 differentially expressed circRNAs were detected in our analysis. Cluster analysis showed that there were significant differences between the experimental group and the control group. We found that 1509 circRNAs were downregulated and 395 were upregulated. Table 1 shows the top 20 differentially expressed circRNAs that were significantly differentially expressed. Among these 20, 10 circRNAs were upregulated and 10 were downregulated. The hits were further thresholded, and those were kept that showed at least a 2-fold change and P-values <0.05. As shown in Figure 2a, the comparison of the two different conditions showed differential expression of circRNAs. Scatter plot results showed robust changes in the expression of circRNAs between the sham control group and the ACI group (Figure 2b). Furthermore, the results of Figure 2c show the distribution of differentially expressed circRNAs in chromosomes. The bar graph based on the gene-derived cyclic ribonucleic acid category shows that 342 exons, 4 antisense, 46 introns, 1 intergene and 13 sense overlapping cyclic ribonucleic acids are upregulated. In contrast, 1301 exons, 2 antisense, 69 introns, 3 intergenes, and 123 sense overlapping cyclic ribonucleic acids were downregulated (Figure 2d).

**Table 1.** Top 10 upregulated and downregulated circRNAs ranked by fold changes after SCI

circRNA	FC (abs)	P-value	FDR	chrom	chrom	strand	circRNA_type	best_transcript	GeneSymbol	GeneSymbol	Regulation
hsa_circRNA_103372	21.04715	0.034768	0.195768	chr3	chr3	-	exonic	NM_016291	IP6K2	IP6K2	up
hsa_circRNA_405965	16.28764	0.012321	0.129885	chr2	chr2	-	exonic	ENST00000392929	CCNT2-AS1	CCNT2-AS1	up
hsa_circRNA_103948	13.66356	0.037862	0.204714	chr5	chr5	+	exonic	NM_021982	SEC24A	SEC24A	up
hsa_circRNA_000102	12.03363	0.012049	0.128922	chr1	chr1	-	intronic	ENST00000357393	AKNAD1	AKNAD1	up
hsa_circRNA_001691	11.66992	0.027711	0.176117	chr7	chr7	-	exonic	NM_002137	HNRNPA2B1	HNRNPA2B1	up
hsa_circRNA_405641	11.58714	0.038655	0.206399	chr17	chr17	-	intronic	ENST00000269392	AZI1	AZI1	up
hsa_circRNA_104939	11.54522	0.04101	0.213631	chr9	chr9	+	exonic	NM_005157	ABL1	ABL1	up
hsa_circRNA_074559	10.41267	0.03915	0.207731	chr5	chr5	-	exonic	NM_016221	DCTN4	DCTN4	up
hsa_circRNA_008389	10.07535	0.012801	0.131943	chr1	chr1	-	exonic	NM_018198	DNAJC11	DNAJC11	up
hsa_circRNA_019744	8.532438	0.039087	0.207485	chr10	chr10	-	exonic	NM_005736	ACTR1A	ACTR1A	up
hsa_circRNA_103887	20.1334	0.00224	0.089408	chr5	chr5	+	exonic	NM_138782	FCHO2	FCHO2	down
hsa_circRNA_101836	19.86489	0.021279	0.159215	chr16	chr16	+	exonic	NM_004555	NFATC3	NFATC3	down
hsa_circRNA_406791	18.30178	0.001674	0.088954	chr6	chr6	+	exonic	NM_015555	ZNF451	ZNF451	down
hsa_circRNA_102995	18.21764	0.004734	0.098011	chr20	chr20	+	exonic	NM_019095	CRLS1	CRLS1	down
hsa_circRNA_102183	16.39273	0.003117	0.092018	chr17	chr17	+	exonic	NM_004459	BPTF	BPTF	down
hsa_circRNA_000581	16.28442	0.001974	0.088954	chr14	chr14	+	exonic	NR_038354	WDR20	WDR20	down
hsa_circRNA_101837	16.22925	0.022342	0.160614	chr16	chr16	+	exonic	NM_004555	NFATC3	NFATC3	down
hsa_circRNA_103572	16.15558	0.010143	0.121564	chr3	chr3	+	exonic	uc003fyk.2	LRCH3	LRCH3	down
hsa_circRNA_101771	14.87513	0.004531	0.097171	chr16	chr16	+	exonic	NM_014494	TNRC6A	TNRC6A	down
hsa_circRNA_092476	14.86703	0.003553	0.092018	chr19	chr19	-	exonic	ENST00000596831	AC004076.9	AC004076.9	down

Finally, we predicted the five most likely potential targets. Table 2 lists the five highest-level candidate circRNAs (the first 10 upregulated and downregulated microRNAs) as binding targets for each circRNA.

**Table 2.** The five highest-ranking miRNA candidates for top 10 upregulated and downregulated circRNAs

circRNA	Predicted miRNA response elements (MREs)				
	MRE1	MRE2	MRE3	MRE4	MRE5
hsa_circRNA_103372	hsa-miR-494-5p	hsa-miR-577	hsa-miR-628-5p	hsa-miR-647	hsa-miR-526b-5p
hsa_circRNA_405965	hsa-miR-4298	hsa-miR-3714	hsa-miR-6072	hsa-miR-6768-3p	hsa-miR-519d-5p
hsa_circRNA_103948	hsa-miR-421	hsa-miR-181a-2-3p	hsa-miR-505-3p	hsa-miR-429	hsa-miR-380-5p
hsa_circRNA_000102	hsa-miR-1296-3p	hsa-miR-194-3p	hsa-miR-3184-3p	hsa-miR-4753-3p	hsa-miR-412-3p
hsa_circRNA_001691	hsa-miR-5193	hsa-miR-6792-3p	hsa-miR-4691-5p	hsa-miR-6509-3p	hsa-miR-4776-3p
hsa_circRNA_405641	hsa-miR-6808-5p	hsa-miR-6764-5p	hsa-miR-4323	hsa-miR-6856-5p	hsa-miR-2861
hsa_circRNA_104939	hsa-miR-193b-3p	hsa-miR-194-3p	hsa-miR-762	hsa-miR-486-5p	hsa-miR-892b
hsa_circRNA_074559	hsa-miR-3942-5p	hsa-miR-4682	hsa-miR-7-2-3p	hsa-miR-8076	hsa-miR-4703-5p
hsa_circRNA_008389	hsa-miR-7111-3p	hsa-miR-6873-3p	hsa-miR-6769b-3p	hsa-miR-760	hsa-miR-1236-3p
hsa_circRNA_019744	hsa-miR-1301-3p	hsa-miR-6812-5p	hsa-miR-650	hsa-miR-4739	hsa-miR-4459
hsa_circRNA_103887	hsa-miR-890	hsa-miR-766-5p	hsa-miR-125a-3p	hsa-miR-10b-3p	hsa-miR-571
hsa_circRNA_101836	hsa-miR-744-5p	hsa-miR-576-3p	hsa-miR-661	hsa-miR-9-5p	hsa-miR-377-5p
hsa_circRNA_406791	hsa-miR-627-3p	hsa-miR-338-3p	hsa-miR-34 c-5p	hsa-miR-4659a-3p	hsa-miR-3688-5p
hsa_circRNA_102995	hsa-miR-424-5p	hsa-miR-16-5p	hsa-miR-195-5p	hsa-miR-15b-5p	hsa-miR-323a-3p
hsa_circRNA_102183	hsa-miR-218-1-3p	hsa-miR-758-3p	hsa-miR-218-5p	hsa-miR-486-5p	hsa-miR-202-3p
hsa_circRNA_000581	hsa-miR-378a-3p	hsa-miR-185-5p	hsa-miR-378d	hsa-miR-422a	hsa-miR-501-5p
hsa_circRNA_101837	hsa-miR-766-5p	hsa-miR-509-5p	hsa-miR-18b-5p	hsa-miR-18a-5p	hsa-miR-380-3p
hsa_circRNA_103572	hsa-miR-29b-2-5p	hsa-miR-544a	hsa-miR-29a-5p	hsa-miR-518 c-5p	hsa-miR-214-3p
hsa_circRNA_101771	hsa-miR-647	hsa-miR-370-3p	hsa-miR-520 c-3p	hsa-miR-526b-3p	hsa-miR-520b
hsa_circRNA_092476	hsa-miR-5007-5p	hsa-miR-760	hsa-miR-4722-5p	hsa-miR-545-3p	hsa-miR-4527

## Validation by qRT-PCR

**Figure 3.**
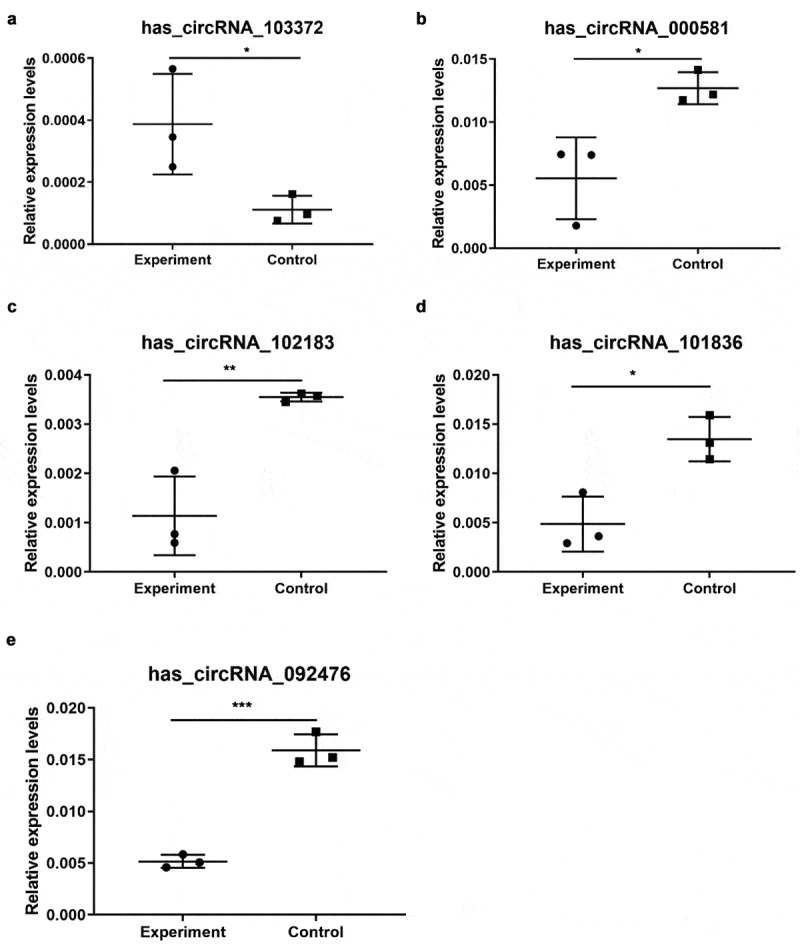
qRT-PCR validation of five selected circRNAs. (a) Relative expression levels of has_circRNA_103372. (b) Relative expression levels of has_circRNA_000581. (c) Relative expression levels of has_circRNA_102183. (d) Relative expression levels of has_circRNA_101836. (e) Relative expression levels of has_circRNA_092476. Compared with the control, rno_circRNA_000581/102,183/101,838/092476 were significantly downregulated. However, rno_circRNA_103372 was significantly upregulated. The data are normalized using the mean ± SEM (*P < 0.05; n = 6 per group)

After analyzing the data by filtering a high-throughput microarray, we listed 10 differentially expressed circRNAs for further analysis and have been able to validate five by qRT-PCR. As summarized in Figure 3, hsa_circRNA_000581, hsa_circRNA_092476, hsa_circRNA_101836, and hsa_circRNA_102183 in the acute cerebral infarction group were significantly downregulated and hsa_circRNA_103372 was significantly upregulated compared to the control group. These results show that the expression patterns of these candidate circRNAs in microarray and PCR data are similar. Table 3 provides a summary on the variation and calculated p-values. The primers used in this study for quantitative reverse transcription polymerase chain reactions are shown in Table 4.

**Table 3.** Comparison of candidate circRNAs expression in microarray and PCR

CircRNAs	Microarray			PCR		
	FC (abs)	*P*-value	Regulation	FC (abs)	*P*-value	Regulation
hsa_circRNA_000581	16.2844189	0.001973896	down	2.287937	0.0238	down
hsa_circRNA_092476	14.8670307	0.003552554	down	3.079344	0.0004	down
hsa_circRNA_101836	19.8648936	0.021279368	down	2.766612	0.0142	down
hsa_circRNA_102183	16.3927337	0.003117049	down	3.118147	0.0066	down
hsa_circRNA_103372	21.0471532	0.034767906	up	3.46493	0.0468	up

**Table 4.** Sequences of primers used for qRT-PCR assay

Gene name	Primer sequence	Ta Opt (**^◦^**C)	Product size (bp)
β-actin (H)	F:5ʹ GTGGCCGAGGACTTTGATTG3’	60	73
	R:5ʹCCTGTAACAACGCATCTCATATT3’		
hsa_circRNA_092476	F:5ʹ AGCAACAGACTCTCCTGCAGA3’	60	135
	R:5ʹ ACTTGCTGCTTGTGTATCCAG 3’		
hsa_circRNA_101836	F:5ʹ CCCATTATGAAACTGAAGGTAGC3’	60	126
	R:5ʹGATGGAGGTGGATCTACATTAAAGA3’		
hsa_circRNA_102183	F:5ʹ TCAAATTCAGGCGTTGTTCAA 3’	60	181
	R:5ʹ TCTGCTGCTCCAGTCGTTTT 3’		
hsa_circRNA_103372	F:5ʹ GTGTGTGTGGCATGCAGC 3’	60	104
	R:5ʹ TGGGAACTTATATTCCCCTTC 3’		
hsa_circRNA_000581	F:5ʹ GCAGGCCAAGTCCAGCTTATA 3’	60	82
	R:5ʹ ACCAACCTCATGGGATTGTTTC 3’

## CircRNAs/MicroRNA/RNA Interaction

**Figure 4.**
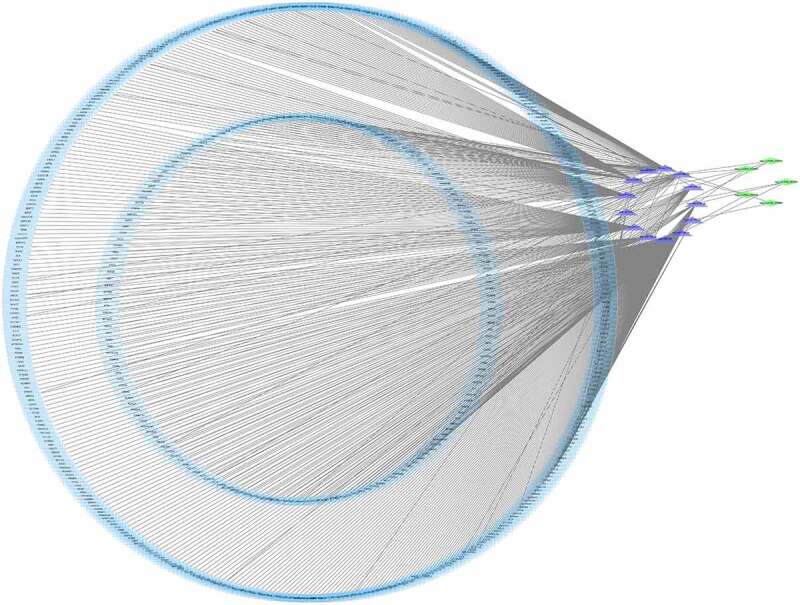
The circRNA/miRNA/mRNA network analysis. The network includes 5 brown nodes circRNAs, 13 red nodes miRNAs and 655 blue nodes mRNAs

Then, five candidate circRNAs verified by RT-PCR were selected, and the possible interacting miRNA targets were further studied using a computational-based prediction approach. Specifically, we have used starbase to predict possible target miRNAs for each circRNA. The criteria for selecting target genes are clipExpNum >3 and pancancerNum ≥3. Finally, 655 target genes, 13 miRNAs and 5 cirRNAs were obtained. Cytoscape results showed that there were five circRNAs, 13 microribonucleic acids, and 655 microribonucleic acids, showing a large interaction network (Figure 4). CircRNAs can be used as the nucleolar component of microRNA, which usually inhibits the target microRNA. Therefore, circRNAs may upregulate target microRNA indirectly by regulating microRNA.

## Go and pathway analysis of putative target genes

Based on these interactions, five circRNA candidates may play an important role in molecular mechanisms by regulating target molecules. Therefore, we performed GO and KEGG analysis on all target genes, which provided strong evidence for further functional verification of these circRNAs.

**Figure 5.**
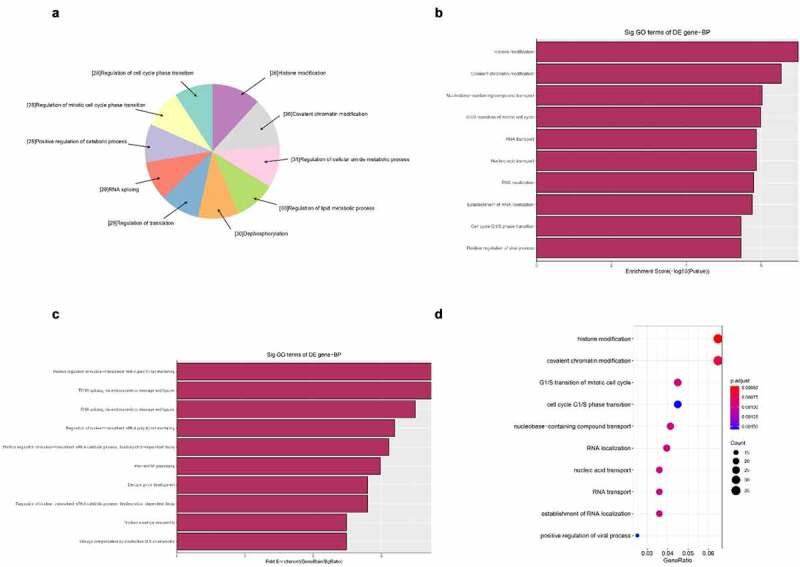
The GO annotations for biological process of target mRNAs regulated by the five candidate circRNAs. Pie (a), bar (b) point charts (c), and dot plot (d) showed the top 10 counts of significant enrichment items

**Figure 6.**
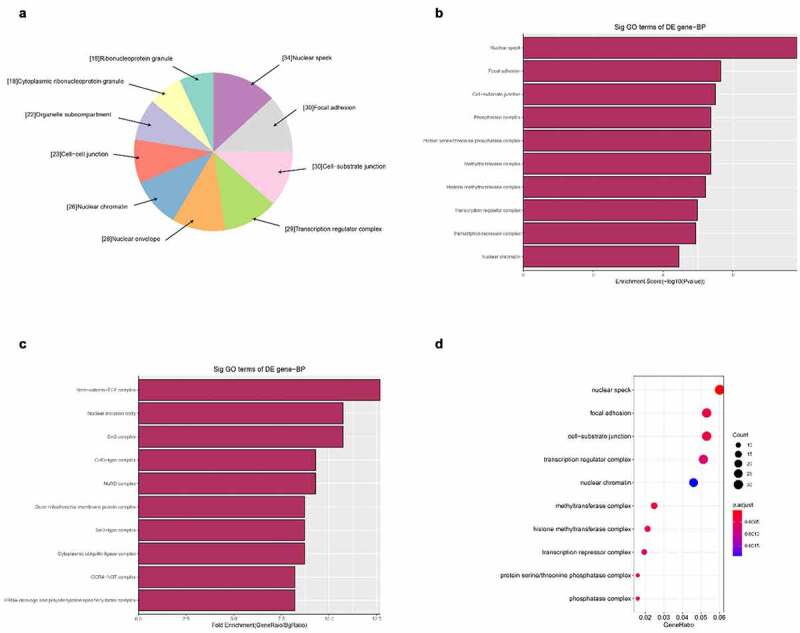
The GO annotations for cellular component of target mRNAs regulated by the five candidate circRNAs. Pie (a), bar (b) point charts (c), and dot plot (d) showed the top 10 counts of significant enrichment items

**Figure 7.**
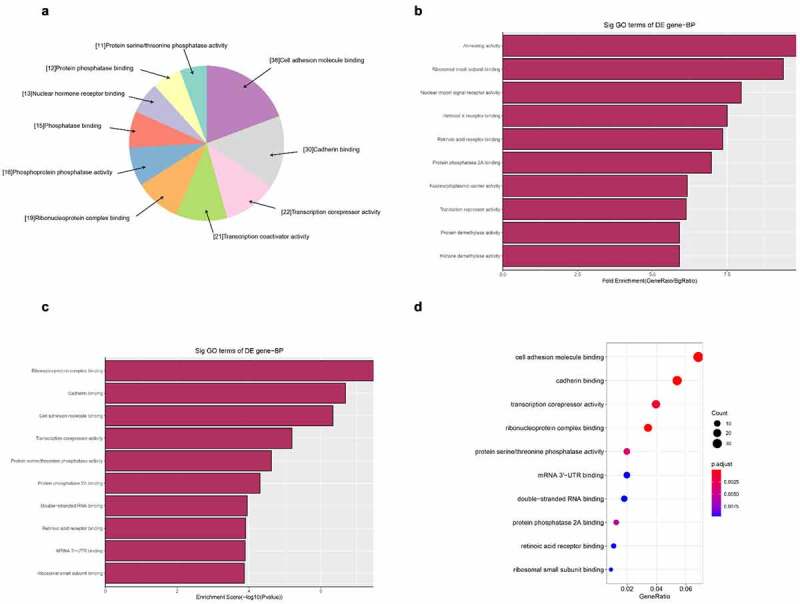
The GO annotations for molecular function of target mRNAs regulated by the five candidate circRNAs. Pie (a), bar (b) point charts (c), and dot plot (d) showing the top 10 counts of significant enrichment items

Next, we carried out GO bioinformatics analysis, including biological processes, cell compositionxs, and molecular function analysis. For each part, we show the following categories of significant enrichment items: top 10 counts, top 10 times enrichment values, top 10 enrichment scores, and gene ratio values. The results show that [1]: The most significant and meaningful terms in biological process are metabolic process and RNA splicing, which describe a series of biological events (Figure 5a-d) [2]. As far as cell components are concerned, the most important enrichment and meaningful terms are intracellular parts and complexes, describing cell components (Figure 6a-d) [3]. As far as molecular function is concerned, the most important enrichment and meaningful term is the binding event, which indicates the function at the molecular level (Figure 7a-d).

**Figure 8.**
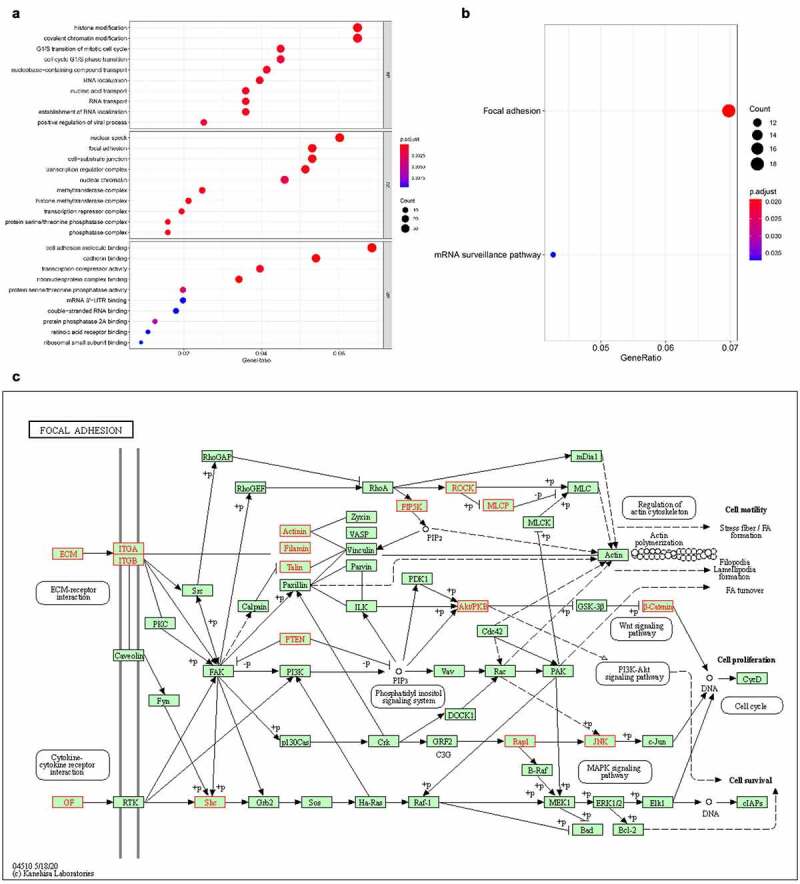
KEGG pathway analysis of five target genes regulated by circRNAs. the bar plot (a), dot plot (b) and related pathway (c) showed the top 10 enrichment score values of the significantly enriched pathway

The top 10 enrichment scores of significant enrichment pathways based on KEGG pathway analysis are shown in Figure 8a. In addition, the dot plot shows the gene ratio values of the top 10 most significant enrichment pathways (Figure 8b). The results showed that these target genes may play an important role in various metabolic activities, and the two most important pathways are the AMPK signaling pathway and the peroxisome-related pathway (Figure 8c).

## Discussion

Cells can resist the accumulation of DNA damage by inducing endogenous DNA repair mechanisms, such as base excision repair (BER), nucleotide excision repair (NER), and non-homologous endpoint ligation (NHEJ) [11,12]. Oxidative DNA damage and repair occur within minutes after ischemic stroke and may last up to 6 months after stroke [13]. DNA repair plays an important role in endogenous brain repair during stroke recovery. DNA repair has a profound impact on a wide range of recovery efforts, including neurogenesis [14,15], angiogenesis [16], axon growth [17], and remyelination [18]. However, homocysteine can play a role in local tissues and promote the generation of oxygen-free radicals, while excess oxygen-free radicals will act on nucleic acids, lipids, and other substances in cells to cause oxidation reaction, resulting in cell damage [19,20]. Oxidized low-density lipoprotein is one of the products of the reaction between lipid and oxygen-free radicals, and its content can indirectly reflect the quantity of oxygen-free radicals and the degree of oxidative stress reaction [21,22]. Therefore, circRNAs may play an important role in the process of cerebral infarction through homologous recombination pathways and may be related to dyslipidemia, hyperhomocysteinemia, and other factors related to the onset of cerebral infarction. The specific mechanism of its action needs to be further explored by designing cell and animal experiments.

In this study, we set out to understand the function of circRNAs in ACI. Our results show that circRNAs are differentially expressed in cerebral infarction, and we identify 10 circRNAs, which are significantly upregulated, and 10 circRNAs, which are significantly downregulated. Our analysis proves that circRNAs can regulate many biological processes, cellular components, and molecular functions. Using RT-qPCR verification, we have been able to verify five downregulated circRNAs. Based on our literature search, we found that HSA _ circrna _ 103,372 (p < 0.01), its source gene UIMC1 is significantly enriched in homologous recombination signaling pathway, which indicates that hsa_circRNA_405965 may participate in ACI by affecting oxidative stress through homologous recombination pathway. At the same time, the miRNAs binding sites on differentially expressed circRNAs and the first five putative target miRNAs bound by differentially expressed circRNAs were identified. Taken together, our data provide an important reference for future research on interaction between circRNA-miRNAs in ACI.

In order to further study the potential functions of differentially expressed circRNAs, we performed a preliminarily analysis by annotating the differentially expressed circRNAs. GO analysis of differentially expressed circRNAs showed that UBXN7, the source gene of hsa_circ_103948, is mainly enriched in ubiquitin binding (GO:0043130) and ubiquitin-like protein binding (GO:0032182). The gene SMARCA5 from hsa_circ_000102 and UIMC1 from hsa_circ_405641 are mainly concentrated in double-strand break repair (GO:0006302) and histone binding (GO:0042393). The source group of hsa_circ_104939 is mainly concentrated in the normal junction of microtube polymerization (GO:0031116), microtube nucleation (GO:0007020), and microtube polymerization or depolymerization (GO:0031112). RSRC1, the source gene of hsa_circ_074559, is mainly enriched in replacing mRNA splicing by splices (GO:0000380). MORC3, the source gene of hsa_circ_008389, is mainly concentrated in maintaining the position of protein in nucleus (GO:0051457), negatively regulating the proliferation of fibroblasts (GO:0048147), and maintaining the location of protein in organelles (GO:0072595). UIMC1 is also rich in active regulation of DNA repair (GO:0045739), repair of broken double strands by non-homologous end connection (GO:0006303), etc. The downregulation of differentially expressed circRNAs may participate in the biological process of acute cerebral infarction and play its function through the above aspects, so we speculate that the downregulation of differentially expressed circRNAs may be related to the growth and repair of neurons. KEGG pathway analysis is not limited to gene level, but a higher-level integration analysis of genes, small biological molecules and proteins is presented as a network information map [15]. By analyzing the source genes of differentially expressed circRNAs, we find that we can better understand their biological pathways and evaluate the possible role of these pathways in cerebral infarction. The enrichment of KEGG biological pathway showed that UIMC1, the source gene of hsa_circ_405641, was significantly enriched in homologous recombination signal pathway (http://www.genome.jp/Kegg-bin/show _ pathway? Hsa03440 + 51,720), and homologous recombination plays an important role in genome maintenance and DNA damage in normal growth cells, which can trigger homologous recombination, which is necessary for cell recovery after oxidative stress [23]. After cerebral infarction, oxidative stress may be an important mechanism to cause a series of secondary damages, which is mainly characterized by the increase of oxygen free radicals and oxidative damage to cell structure [24,25], which will lead to the damage of many components including protein, lipid and DNA [26]. Oxidative DNA damage is one of the most harmful consequences of increased oxidative stress in ischemic stroke. When DNA damage is not repaired, it can trigger a variety of death-promoting signaling pathways, which can induce apoptosis and endanger the functional recovery after stroke. However, CircRNAs, a non-coding RNA molecule with multiple biological functions, exists stably in various body fluids and exosomes, and participates in regulating the occurrence and development of various diseases [27]. Studies have shown that the expression profile of circRNAs in brain tissue will change when the brain tissue lacks blood supply, and some circRNAs may participate in the process of cerebral ischemia [28]. In order to explore the potential biological function of differentially expressed circRNAs in the pathogenesis of acute cerebral infarction, this experiment entrusted Shanghai Yunxu Company to support high-throughput sequencing, and provided enrichment analysis of GO function and KEGG signaling pathway by bioinformatics methods. Sequencing results showed that compared with the control group, there were many differentially expressed circRNAs in patients with ACI, which was statistically significant. In this study, we detected 15 downregulated and 3 upregulated circRNAs significantly after cerebral infarction, and these differentially expressed circRNAs may be involved in the occurrence and development of cerebral infarction. Studies have shown that there are many circRNAs expressions in brain tissue, which are widely involved in the regulation of nervous system diseases and the occurrence and development of cardiovascular diseases related to vascular endothelial function [29]. A recent study showed that there were significant differences in the expression of specific circRNAs in brain tissue under ischemia stimulation. In ischemia reperfusion injury model, compared with the control group, the expression of 15 kinds of circRNAs in hippocampus of mice in transient ischemic group changed, among which 3 kinds increased and 12 kinds decreased. Some studies suggest that these circRNAs may play a role as molecular sponges of miRNAs in the process of hypoxic-ischemic brain damage. The upregulated target miRNA of circRNA-015947 were predicted by using prediction software, and the top five predicted values were miR-188-3p, miR-329-5p, miR-3057-3p, miR-5098 and miR-683. These miRNAs have been proved to be involved in the regulation of the pathogenesis of ischemic brain injury [30]. Recent studies have found that after traumatic brain injury (TBI), 8036 circRNAs changed, among which 16 changed significantly (5 were upregulated and 11 were downregulated). Circ RNA chr8 _ 87, 859, 283–87, 904, 548 significantly increased about 4 times in the cerebral cortex around the injured site, and CXCR2 protein was increased by sponge mmu-let-7a-5p to promote neuroinflammation. The results indicated that the increased circrna chr8 _ 87, 859, 283–87, 904, 548 blocked the recovery of nerve function after TBI [31].

At present, most of the research studies on circular RNA related to cerebral infarction are aimed at the cellular level. This study uses the plasma of patients with acute cerebral infarction in clinical practice, which has more practical and specific functions and mechanisms for the subsequent research on circular RNA related to cerebral infarction. Detailed animal experiments and functional verification are carried out, in order to clarify the role of these circRNAs in ACI and study the functional effects of specific circRNA-miRNA interactions. The specific functions of these significantly differentially expressed circRNAs found in this study have not been experimentally explored.

## Limitations

There are relatively few samples in this sequencing test, and the sample selection time is slightly single. Although the mixed samples are used in early sequencing and the individual errors can be reduced to a certain extent, the mixed samples will cover up the differential expression among some individuals, resulting in experimental results. There may be bias; Patients with acute cerebral infarction are not grouped according to the location, area, and severity of lesions, which can make the differentially expressed circRNAs more specific; the specific functions of these significantly differentially expressed circRNAs found in this study are still unclear.

## Future directions

At present, most of the research studies on circular RNA related to cerebral infarction are aimed at the cellular level. This study uses the plasma from patients with acute cerebral infarction in clinical practice, which has more practical and specific functions and mechanisms for the subsequent research on circular RNA related to cerebral infarction. Detailed animal experiments and functional verification are carried out, in order to clarify the role of these circRNAs in acute cerebral infarction and study the functional effects of specific circRNA-miRNA interactions. In the future, we will further explore the guiding significance of these circRNAs in acute cerebral infarction based on previous work.

## Conclusions

In summary, our results suggest that there are 10 differentially expressed circRNAs after acute cerebral infarction, which may upregulate target microRNA indirectly by regulating microRNA.

## Supplementary Material

Supplemental Material

## Data Availability

The authors confirm that the data supporting the findings of this study are available within the article. https://pan.baidu.com/s/1RbY4bcv8Bu2jiy2YKKp1HA

## Highlights

- CircRNAs show differential expression after acute cerebral infarction.
- circRNAs may upregulate target microRNA indirectly by regulating microRNA.
- hsa_circRNA_000581, hsa_circRNA_092476, hsa_circRNA_101836, and hsa_circRNA_102183 are significantly downregulated, and hsa_circRNA_103372 is significantly upregulated in ACI
- Target genes of the validated circRNAs play an important role in metabolic processes of the cell.
