# Supplementary material for: Circular RNA expression alteration and bioinformatics analysis in patients with acute cerebral infarction injury
Source: Bioengineered. 2021 Dec 7;12(2):11490–505. doi: 10.1080/21655979.2021.2009960 (PMC8810197; doi:10.1080/21655979.2021.2009960)
Supplement: Supplemental Material [file KBIE_A_2009960_SM7551.zip › supplementary/supplementary file flow sheet abstract.docx]

conclusion

In summary,our results suggest that there are 10 differentially expressed cyclic ribonucleic acids after acute cerebral infarction, which may up regulate target microRNA indirectly by regulating microRNA.

Results

Screening and identification

The cyclic ribonucleic acid/ribonucleic acid interacting with microribonucleic acid was constructed by bioinformatics tools

GO and pathway analysis of putative target genes

The accuracy of the data were verified by qRT-PCR

The differential changes of plasma circRNAs expression in patients with acute cerebral infarction by high-throughput sequencing technique

patients with normal physical examination(n=3)

acute cerebral infarction patients(n=3)
