# Supplementary material for: Circular RNA expression alteration and bioinformatics analysis in patients with acute cerebral infarction injury
Source: Bioengineered. 2021 Dec 7;12(2):11490–505. doi: 10.1080/21655979.2021.2009960 (PMC8810197; doi:10.1080/21655979.2021.2009960)
Supplement: Supplemental Material [file KBIE_A_2009960_SM7551.zip › supplementary/supplementary file infographic abstract.docx.veyx2fl.partial]

**Hierarchical cluster analysis**


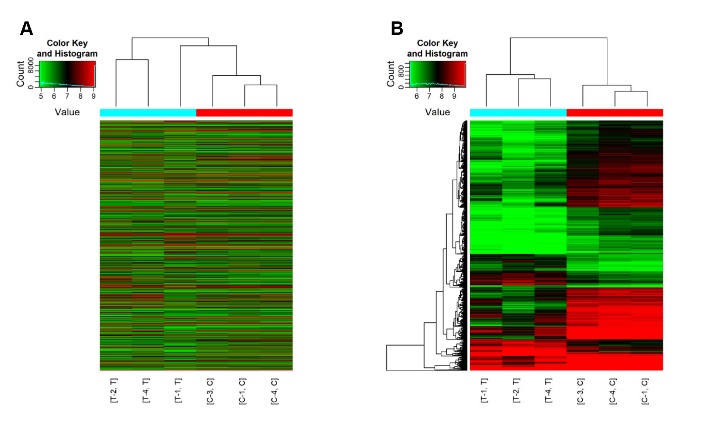


**Expression pattern of cyclic ribonucleic acid**

**
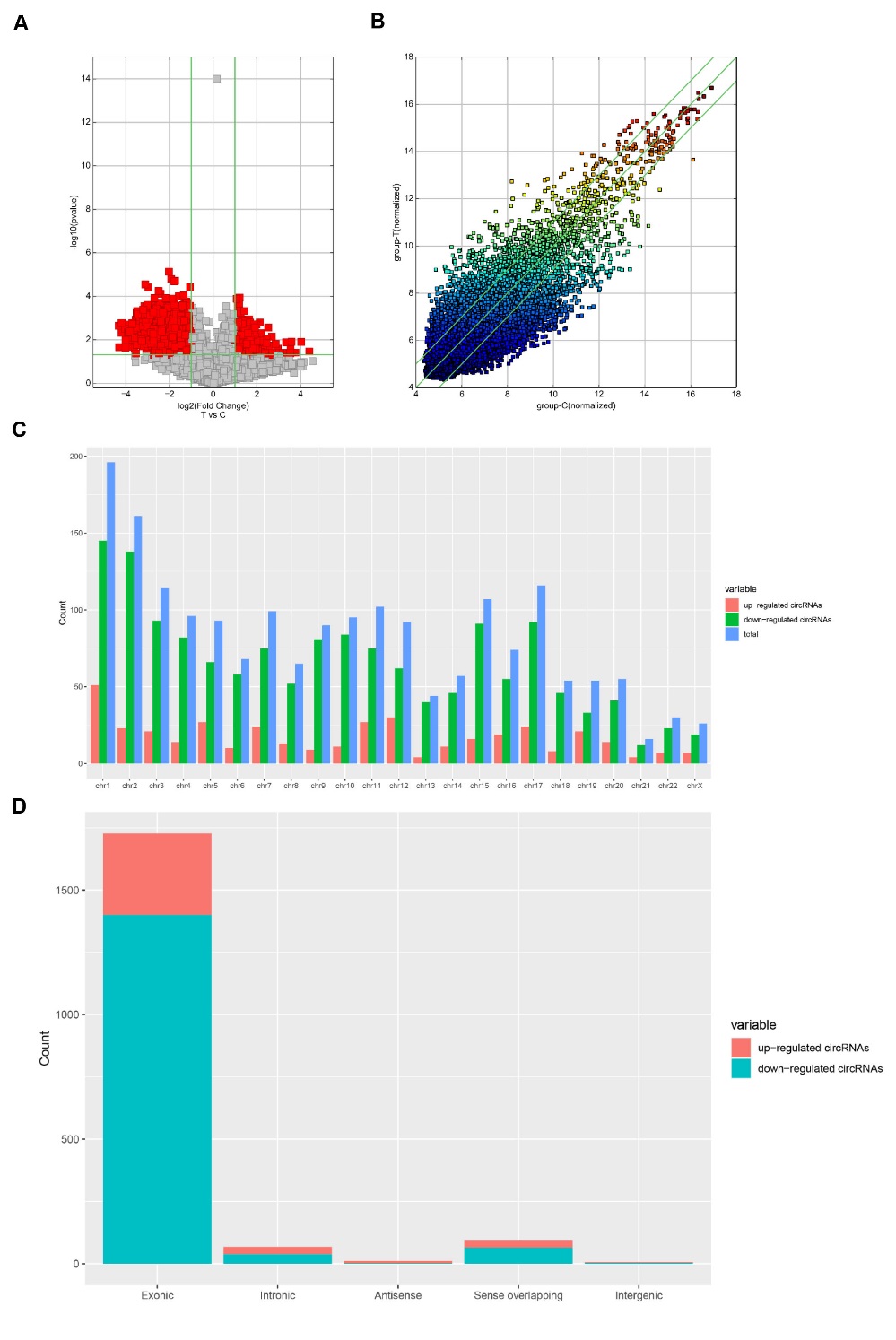
**

**Validation by** **qRT-PCR**

**
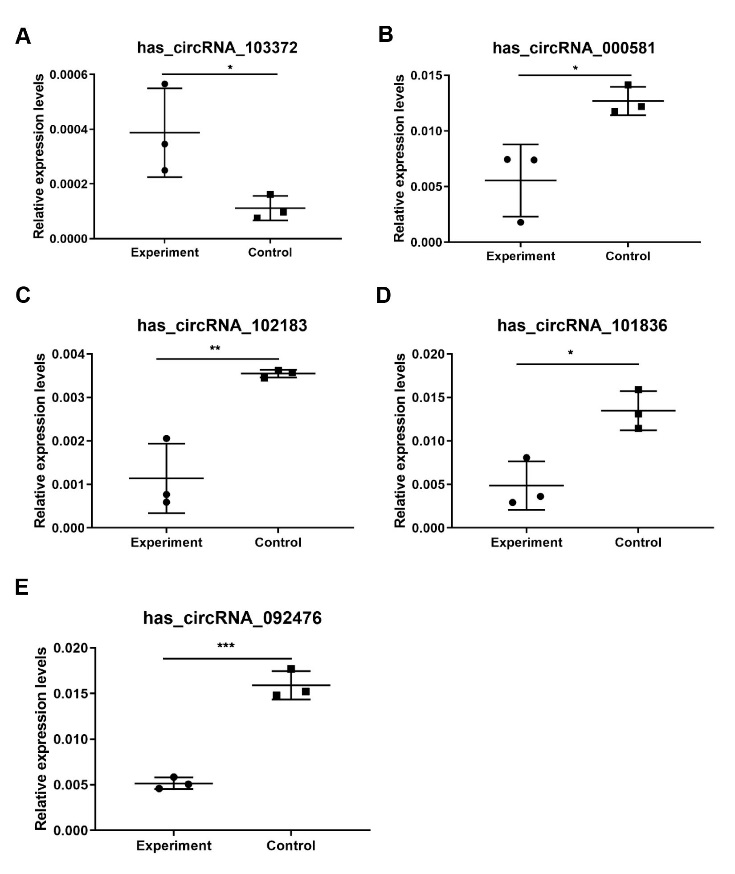
**

**The circRNA/miRNA/mRNA network analysis**

**
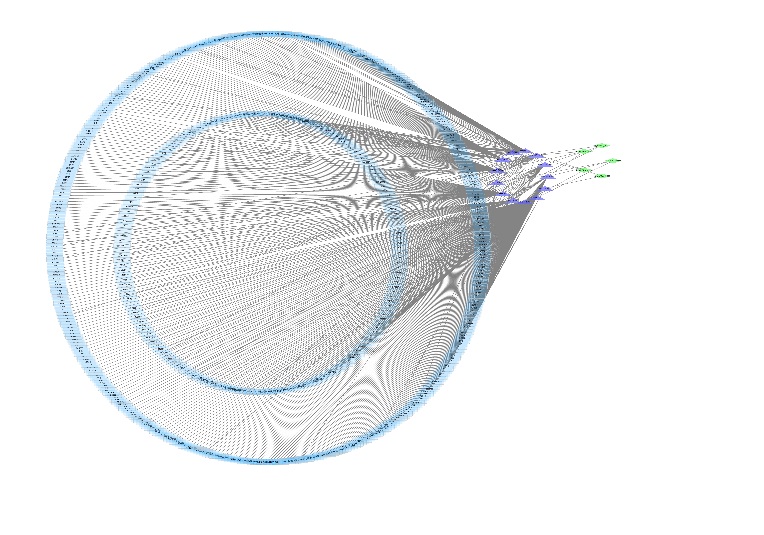
**

**GO and pathway analysis of putative target genes**


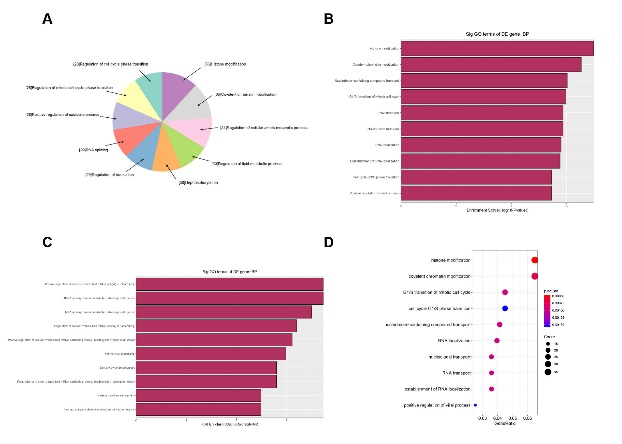

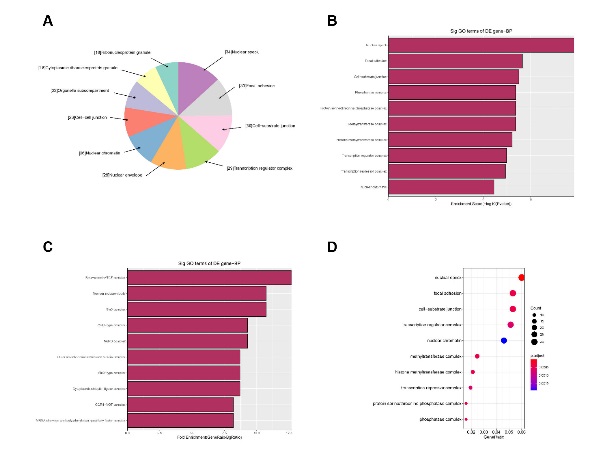


**
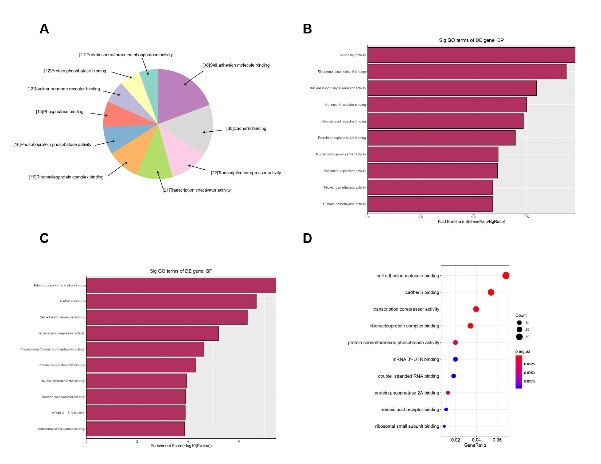

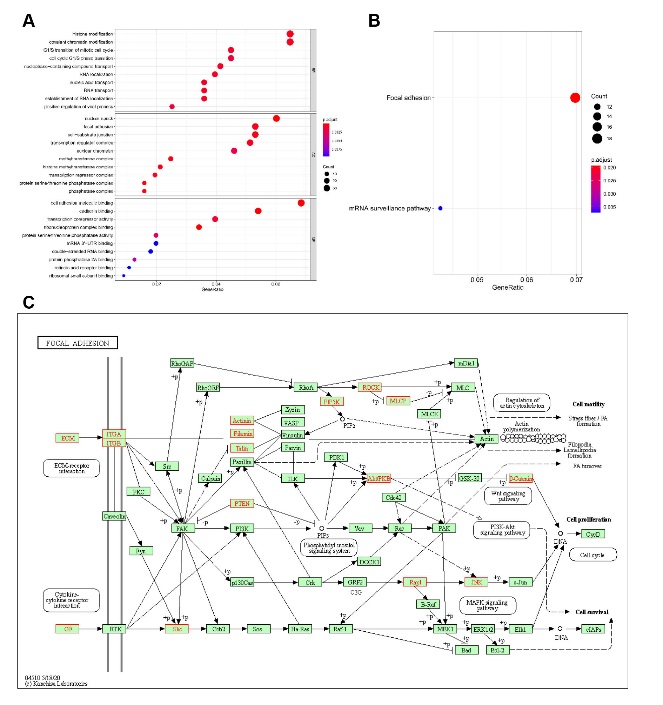
**

**Circular RNA Expression Alteration and Bioinformatics Analysis in Patients with Acute Cerebral Infarction Injury**
