# Supplementary figures and images for: Circular RNA expression alteration and bioinformatics analysis in patients with acute cerebral infarction injury
Source: Bioengineered. 2021 Dec 7;12(2):11490–505. doi: 10.1080/21655979.2021.2009960 (PMC8810197; doi:10.1080/21655979.2021.2009960)

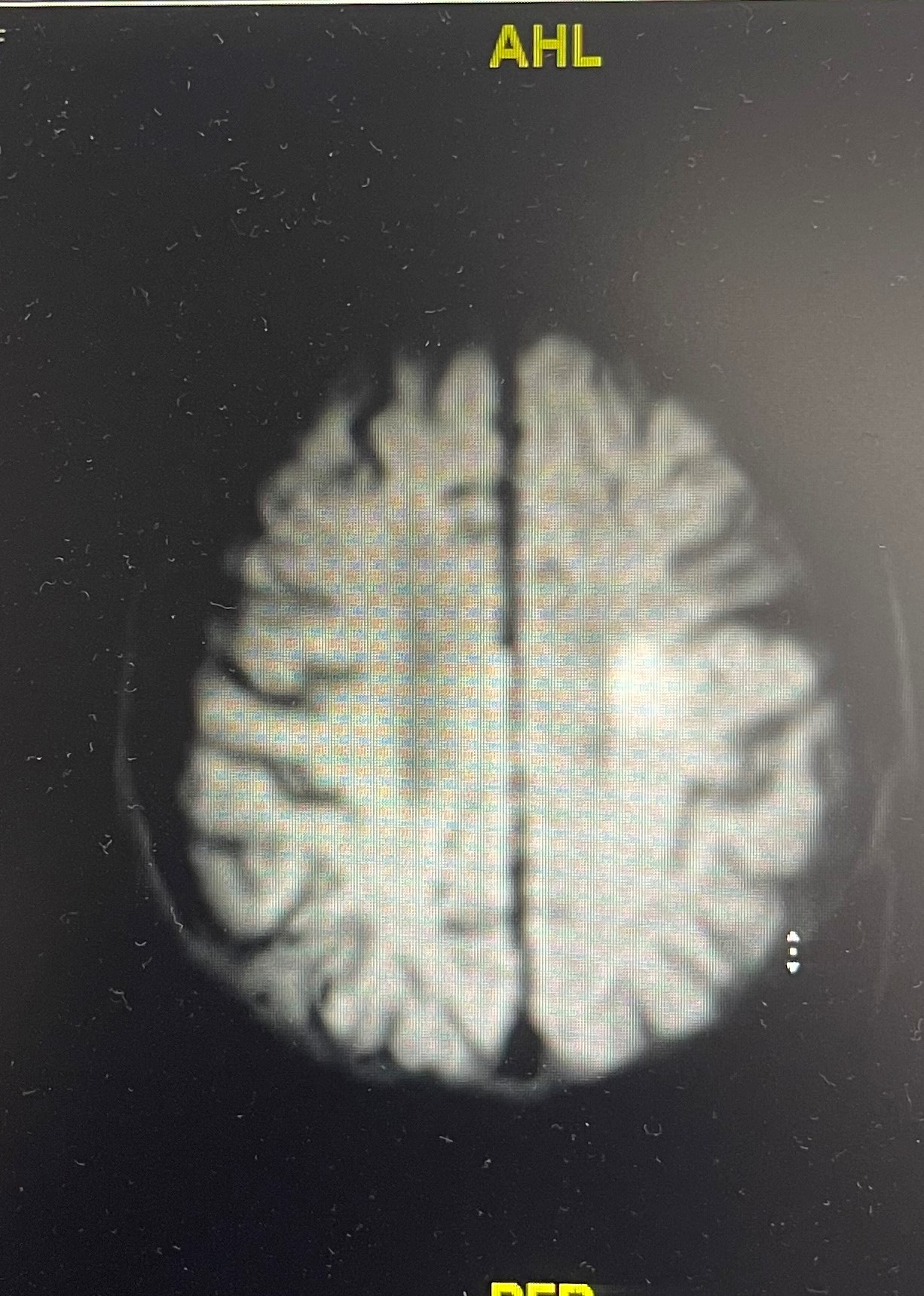

Supplement: Supplemental Material [file KBIE_A_2009960_SM7551.zip › supplementary/Supplementary Figure 1.jpg]

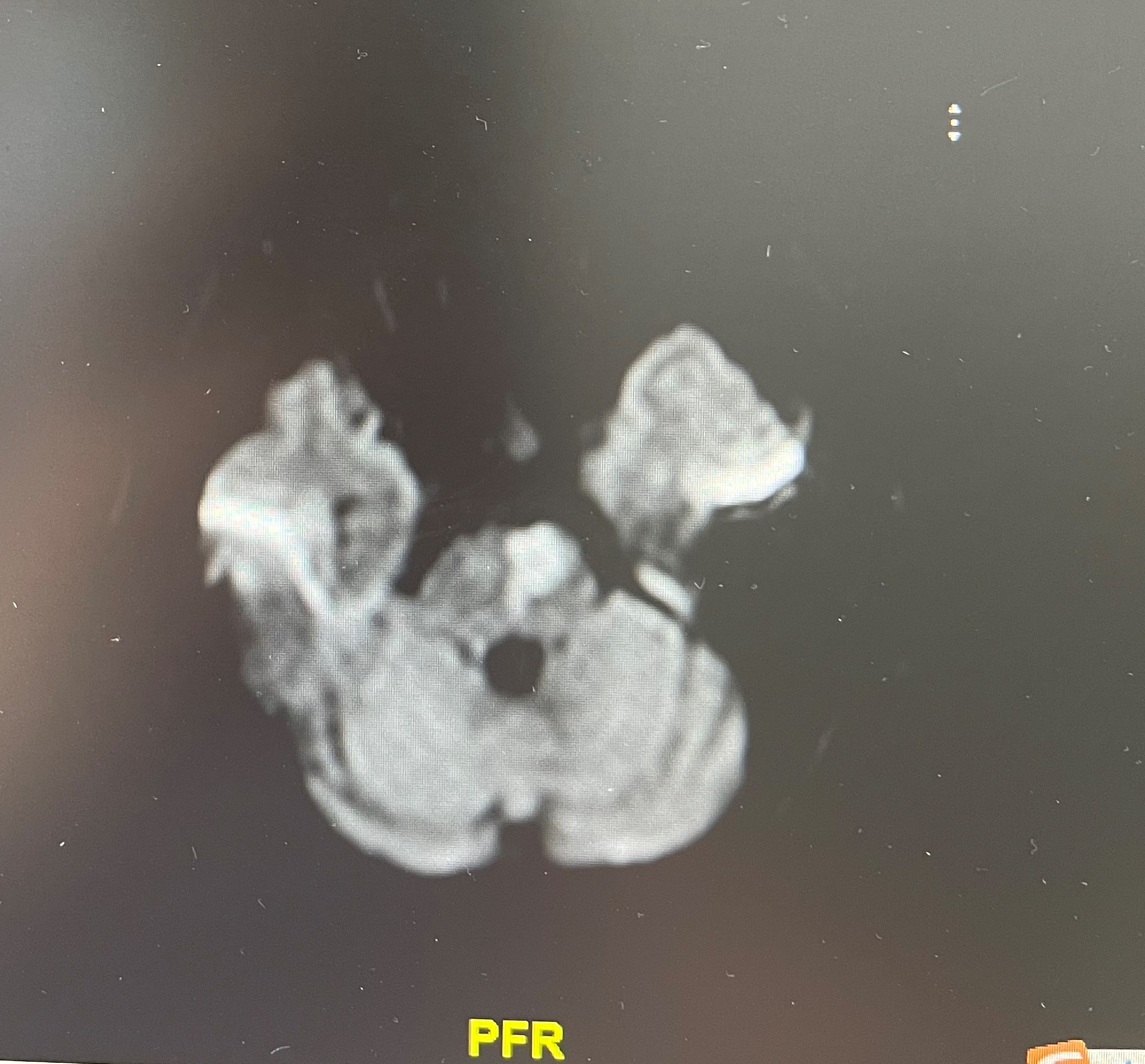

Supplement: Supplemental Material [file KBIE_A_2009960_SM7551.zip › supplementary/Supplementary Figure 2.jpg]

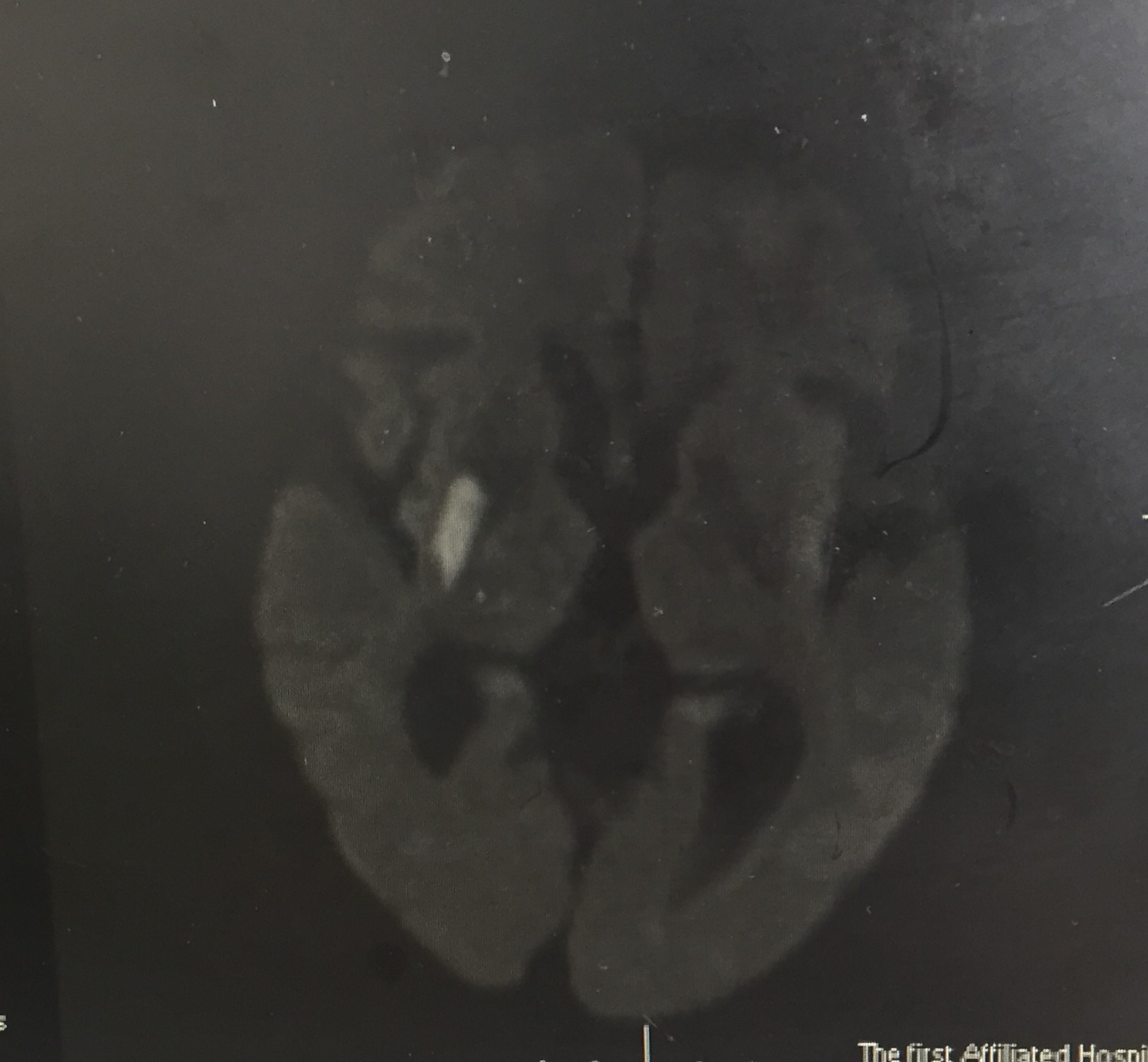

Supplement: Supplemental Material [file KBIE_A_2009960_SM7551.zip › supplementary/Supplementary Figure 3.jpg]
